# Achieving good‐quality consent: review of literature, case law and guidance

**DOI:** 10.1002/bjs5.50306

**Published:** 2020-05-31

**Authors:** P. Normahani, V. Sounderajah, W. Harrop‐Griffiths, A. Chukwuemeka, N. S. Peters, N. J. Standfield, M. Collins, U. Jaffer

**Affiliations:** ^1^ Imperial Vascular Unit London UK; ^2^ Anaesthetic Department London UK; ^3^ Department of Cardiothoracic Surgery London UK; ^4^ Connected Care Bureau Imperial College NHS Healthcare Trust London UK; ^5^ Department of Surgery and Cancer London UK; ^6^ National Lung and Heart Institute Imperial College London London UK; ^7^ London Borough of Hounslow Council London UK

## Abstract

**Background:**

Informed consent is an integral part of clinical practice. There is widespread agreement amongst health professionals that obtaining procedural consent needs to move away from a unidirectional transfer of information to a process of supporting patients in making informed, self‐determined decisions. This review aimed to identify processes and measures that warrant consideration when engaging in consent‐based discussions with competent patients undergoing elective procedures.

**Methods:**

Formal written guidance from the General Medical Council and Royal College of Surgeons of England, in addition to peer‐reviewed literature and case law, was considered in the formulation of this review.

**Results:**

A framework for obtaining consent is presented that is informed by the key tenets of shared decision‐making (SDM), a model that advocates the contribution of both the clinician and patient to the decision‐making process through emphasis on patient participation, analysis of empirical evidence, and effective information exchange. Moreover, areas of contention are highlighted in which further guidance and research are necessary for improved enhancement of the consent process.

**Conclusion:**

This SDM‐centric framework provides structure, detail and suggestions for achieving meaningful consent.

## Graphical abstract

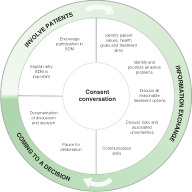

A strategy for shared decision‐making

## Abstract (es)

**Antecedentes:**

El consentimiento informado es una parte integral de la práctica clínica. Existe un acuerdo generalizado entre los profesionales de la salud en que lograr el consentimiento del procedimiento no debe ser una transferencia unidireccional de información, sino un proceso de apoyo a los pacientes en la toma de decisiones informadas y autodeterminadas. Esta revisión tiene como objetivo identificar procesos y medidas que deban ser considerados al hablar sobre el consentimiento con pacientes autosuficientes sometidos a procedimientos quirúrgicos electivos.

**Métodos:**

Al planear esta revisión se tuvo en cuenta la recomendación formal por escrito del Consejo Médico General y del *Royal College of Surgeons of England*, además de la literatura revisada por pares y de la jurisprudencia.

**Resultados:**

Se presenta un marco para lograr el consentimiento que se basa en los principios clave de la toma de decisiones compartida (*Shared Decision‐Making*, SDM); un modelo que aboga por la contribución, tanto del médico como del paciente, al proceso de toma de decisiones a través del énfasis en la participación del paciente, el análisis de la evidencia empírica y el intercambio efectivo de información. Además, se destacan áreas de contención en las que se necesitan más recomendaciones y más investigación para mejorar aún más el proceso del consentimiento.

**Conclusión:**

Este marco centrado en la SDM proporciona estructura, detalles y sugerencias sobre cómo se puede lograr un consentimiento informado satisfactorio.

## Introduction

There has been a paradigm shift in the approach by which consent for procedures is obtained by health professionals. Despite movement away from the patriarchal approach of yesteryear, inadequate consenting practice continues to be flagged as a prevalent medicolegal issue^1^, ^2^, ^3^. In response, in the UK shared decision‐making (SDM) has emerged as a vital model to incorporate in achieving good‐quality consent^4^.

SDM^5^ is a model that encourages health professionals to collaborate with patients for decision‐making purposes through the use of empirical evidence alongside a tailored patient‐centric approach^6^. In practice, SDM aims to facilitate clinician–patient dialogues, particularly when evaluating multiple treatment options, whilst respecting the patient's subjective weighting of values and specific treatment outcomes. Patients' perception of having participated in SDM has been demonstrated to improve outcomes such as satisfaction, decisional conflict and patient‐reported health outcomes^7^.

SDM has gained greater mainstream coverage in recent years following the high‐profile validation of its core tenets in the landmark Montgomery *versus* Lanarkshire case^8^. This case centred around the failure of the clinical team to convey the risk of shoulder dystocia to a pregnant diabetic patient (Nadine Montgomery), a risk that notably affects 10 per cent of vaginal deliveries in diabetic mothers. Of key importance, this information was deliberately withheld in consultations for fear of provoking patient anxiety. In response, the judge ruled that there is a duty of care on behalf of medical professionals to discuss such procedural ‘material risks’. Most strikingly, this case highlighted the pressing need for health professionals to challenge their consenting practice.

**Figure 1:**
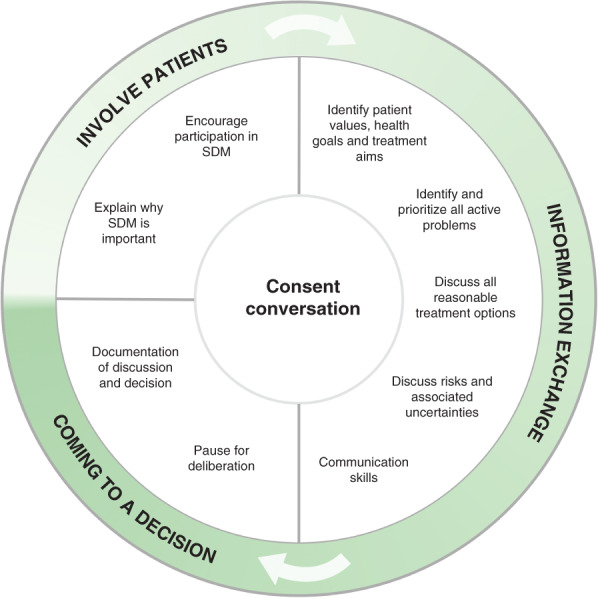
Proposed shared decision‐making consent model SDM, shared decision‐making.

This review proposes a consenting framework for health professionals (*Fig*. 1) that synthesizes processes put forward by the SDM model as well as contemporary guidance from case law, governing bodies – the General Medical Council (GMC) and the Royal College of Surgeons of England (RCS) – and peer‐reviewed literature. It focuses specifically on obtaining procedural consent from competent patients undergoing elective procedures, as this cohort constitutes the vast majority of a procedure‐centric health professional's caseload.

## Importance of engaging patients

Engaging patients is the crucial step in starting consent‐related discussions. Interestingly, Pollard and colleagues^9^ suggested that clinicians are often sceptical about their patients' ability and interest in making health‐related decisions, although these assumptions are often inconsistent with patients' stated preferences^10^, ^11^, ^12^, ^13^. In contrast, Joseph‐Williams *et al*.^14^ suggested that the root of poor patient engagement is rarely due to a lack of interest and is multifactorial – a result of the information asymmetry inherent to the clinician–patient dialogue and patients' lack of awareness of their importance within the encounter.

In fact, Chewning and co‐workers^11^ found that 75 of 119 studies analysed as part of a systematic review reported that the majority of patients prefer to participate in key decisions. This stated preference was even higher when making treatment decisions regarding invasive procedures, with 11 of 14 studies reporting that the majority of patients would like to be involved in decision‐making.

Observational analysis from a pilot programme incorporating SDM in clinical encounters across the UK suggested that clinicians can play an important role in improving patient engagement by simply explaining why SDM is important and encouraging participation^4^.

## Facilitating information exchange

The crux of SDM is the effective exchange of information between patients and health professionals. Achieving this is a multifaceted and difficult process^6^.

### Identification of patients' values and goals

The identification of values and goals in the consent process has been identified as an important part of SDM models^5^, ^6^, ^15^. It is of particular importance in complex care situations, for instance with older multimorbid patients. Reuben and Tinetti^16^ have argued that the focus should be on the patient's most pressing issues rather than their ‘presenting complaint’. The patient's individual health goals (such as prolongation of life, functional independence), values (for instance, outlook on life, spirituality, religion) and their treatment aims (such as cure, improved quality of life, improved functionality, no treatment, symptom control) should be discussed in order to formulate personalized treatment options^15^, ^16^. This goal‐oriented approach has the advantage of simplifying decisions for people with multiple conditions by focusing on outcomes that are a priority to them.

### Exploration of all management options

The core ethical principles of autonomy and right to self‐determination deem it imperative formally to discuss the choice between all viable treatment options with patients.

Presenting a clear list of these options, side by side, has been shown to provide a good structure for discussion^17^, ^18^. Discussion of options should include a simple description of the treatments, an explanation of any clear differences between them (for example, surgical *versus* medical), any potential risks and benefits, and the likelihood of success. The use of positive terms such as ‘active surveillance’ is favourable when describing non‐surgical options in order to avoid ‘framing manipulation’, in which people react to a particular choice in different ways depending on how it is presented^17^. An interesting scenario arises in considering a potentially superior intervention that may be unavailable from the clinician or institution. In these situations, best practice would be to involve colleagues who are competent in this technique rather than to defer to the more readily available option.

Moreover, for high‐risk procedures, it is particularly important to anticipate and discuss plausible postoperative scenarios, such as admission to an intensive care setting or further intervention whilst incapacitated. Pecanac and co‐workers^19^, ^20^ observed 48 discussions between patients and surgeons regarding high‐risk surgery. They observed that surgeons described the gravity of the operation and proceeded with conversations as if ‘buy‐in’ for prolonged life support or further procedures had already been established, even though this had rarely been agreed explicitly.

### Communication of risk

Effective communication of risk is another integral part of obtaining informed consent; however, it is an extremely challenging and complex task that is often performed poorly by clinicians^4^.

Risk can take on a number of different forms, including side‐effects, complications, and failure of an intervention to achieve the desired outcome. The GMC advises discussion of less serious risks or complications if they occur frequently, and discussion of more serious risks even if the likelihood is very small^21^. Additionally, the GMC advises against the withholding of any information from patients for any reason if that information is necessary for decision‐making, unless there are exceptional circumstances in which it may cause serious harm^21^.

The case of Montgomery *versus* Lanarkshire Health Board builds further on the groundwork laid out by the GMC guidance, by addressing the topics of ‘material risk’ and the now defunct ‘Bolam principle’. Adequacy of patient consent is no longer determined by whether the disclosure would be deemed acceptable by a responsible body of the medical profession. Instead, a patient‐centred test should be applied to ensure that the health professional has ‘taken reasonable care to ensure that the patient was made aware, before consenting, of any material risks, and of any reasonable alternative or variant treatments’. Here, the test of materiality is whether, ‘in the circumstances of the particular case, a reasonable person in the patient's position would be likely to attach significance to the risk, or the doctor is or should reasonably be aware that the particular patient would be likely to attach significance to it’^22^.

The position of the ‘reasonable person’ may be understood broadly as the essential information required by all patients in a certain treatment setting, whereas the position of the ‘particular patient’ refers to the obligation of doctors to tailor risk information around the values that the particular patient holds^23^.

The case of Mrs A *versus* East Kent Hospitals University NHS Foundation Trust (2015) reassuringly signifies that the law does recognize sound reasoning when applying the test of a ‘reasonable patient’^8^. In this case, the claimant argued that there had been a failure to detect a chromosomal abnormality (risk of 1 in 1000) during her pregnancy following *in vitro* fertilization. The court applied the Montgomery test and decided that the risk was not material as the claimant was prepared to accept the background risk of having a disabled child, having *de facto* accepted that risk in relation to a test for Down syndrome (risk of 1 in 1753).

The need to assess individual circumstance when conveying risk is also paramount. This was discussed by James Badenoch QC in the Montgomery case^24^; he explained that the probability of a risk is not a decisive measure of whether it requires disclosure. That is to say, a serious risk, however rare, may be of particular significance to a patient whose livelihood or life may be especially affected if the risk were to occur. Rogers *versus* Whitaker (High Court of Australia, 1992)^25^ *,* as cited in the Montgomery case*,* was an instance where the plaintiff was offered an operation on her blind eye. The operation carried an unexplained 1 in 14 000 risk of ‘sympathetic ophthalmia’, which occurred and resulted in the loss of sight in her one functional eye, thus leaving her blind^24^. The judge acknowledged that, although a risk this small may not regularly require disclosure, in this particular context a ‘reasonable person’ would likely attach significance to injury to their one remaining functional eye.

There is also ongoing debate surrounding procedures that house exceptionally rare, but potentially debilitating, procedural risks. Determining whether risks of this magnitude are material to treatment decisions is challenging and requires further guidance.

When discussing risks, it is important to recognize that patients' subjective understanding of risk can be vastly different from that of the healthcare professional. This view is supported by a study of 71 patients waiting for a carotid endarterectomy who were surveyed regarding their understanding of the risk of stroke associated with surgery^26^. It was found that patient estimation of risk differed significantly from what they had been told (estimates ranged from 0 to 65 per cent, whereas the actual risk is 2 per cent).

As demonstrated, it is important to convey risk information in a clear and precise manner. Such strategies include:

- Avoiding descriptive terms (such as ‘high risk’) as these reflect the doctor's perspective and may be misinterpreted by the patient^27^, ^28^.
- The use of numbers to represent the probability of risk provides a more accurate understanding^29^. Using both absolute (such as 1 in 100) and relative risks (for example, 3 times more common) together has been shown to be the optimal option^27^.
- When using numbers there is strong evidence for the use of a consistent format (for instance, 1 in 100 or percentage values), as well as a consistent denominator^28^, ^29^, ^30^.
- Individualized risk, as weighted by patient‐specific co‐morbidities and functional status, has been shown to be more valuable than population data^21^, ^27^, ^29^. With growing public awareness of institution‐ and surgeon‐specific risk, the authors anticipate that these figures will require incorporation into the consent process in the near future.
- Patients often find it difficult to achieve a sense of scale, especially when risks are not common^27^. The use of ‘real world’ comparisons (such as the risk of a road traffic accident) helps to add perspective^28^.
- Visual aids such as graphs can help in understanding probabilities, particularly for patients with lower numeracy skills^27^, ^28^. This is particularly so if these tools are structured and interactive^29^. However, some patients find extracting information from graphs challenging^30^.

There are certain patients who are unwilling to engage in dialogue regarding procedural risk. The legal and GMC best practice stance is that any reasons for refusal should be explored and if, after discussion, a patient still wishes not to know in detail about the planned procedure, their wishes should be respected as feasibly possible^21^, ^22^. However, it is important to note that withholding certain important details of the procedure, even at the wish of the patient, may invalidate the consent^21^, ^24^.

### Communication of uncertainty

Communication of uncertainty is similarly challenging, but essential for informed decision‐making. Uncertainty can relate to the randomness of future events or to the ambiguity of the risk information that is available. Studies on how best to communicate uncertainty are lacking, and the best strategy remains uncertain^30^.

Interpretation of risk by patients is not dependent solely on the probability of the risk, as discussed above, but also on an understanding of the potential harm that may result. For example, a patient undergoing carotid endarterectomy may be given information about the probability of perioperative stroke, but may undervalue the potential harm, because their only experience of a stroke may have been when a friend developed only minor symptoms. It is therefore advised that the uncertain but potential impact of a complication on patients should be clarified.

### Communication skills

Good‐quality consent must be built on robust communication skills. Health professionals must be able to tailor their approach to the patient's needs, and to the nature and level of risk involved. Core communication skills such as using clear, simple and consistent language, building rapport, generating dialogue, signposting and active listening are vital.

Issues that may impair effective communication with the patient, such as hearing, eyesight, literacy, pain and anxiety, should also be taken into account. If required, additional support should be provided to maximize understanding of information^18^, ^21^. This may include using an advocate, an interpreter, provision of an audio recording or a written record and use of advocacy services such as support groups^18^, ^21^.

## Use of decision aids in facilitating information exchange

A recent Cochrane review^31^ of studies investigating the effect of adjunctive decision‐making tools in more than 30 000 patients found that the use of decision aids (DAs) improves patient knowledge, leads to more accurate expectations about the risks and benefits associated with different options, and results in patients making decisions consistent with their preferences, values and goals.

DAs must be evidence‐based, up‐to‐date and patient‐centred. Some authors^32^ have suggested a systematic approach to identifying ‘core information’ using a mixed‐methods approach that involves collating relevant information from multiple sources.

There are at least two categories of DA that can be used to promote SDM: patient decision aids (PDAs) and conversational aids^33^. PDAs, which are available in a range of media formats (online, print, video), are often used outside consultations and aim to improve patient knowledge and encourage their involvement in decision‐making. Conversational aids are used to encourage and enhance discussions between patients and clinicians. A notable feature of DAs is that they allow the individual to consider their values when making decisions. Most DAs aim to achieve this implicitly by clarifying the pros and cons of treatment options. Others achieve this in more explicit ways, such as ranking and weighting different features of treatment options to facilitate a decision^34^.

Some have warned against overreliance on PDAs, which may promote autonomous decision‐making rather than SDM^33^. Furthermore, PDAs demand significant work from patients before consultations, and there is a risk that clinicians assume that those who have used them no longer need to engage in dialogue. Conversational aids seem to promote clinician–patient interactions consistent with SDM, and are therefore often preferred^4^, ^35^.

## Arriving at a decision

To give the patient enough time to reflect and to consult friends and family, more than one discussion may be required before arriving at a shared decision^17^.

Guidance from the RCS^18^ and the GMC^21^ indicates that a clinician may recommend a particular option that they believe is best for the patient as long as they remain impartial and do not put pressure on the patient to accept their advice. However, complexity lies within such recommendations, as the clinician's well intentioned advice may well be based upon a myriad of factors (such as what is more successful in their hands, more enjoyable to perform, provides better reimbursement). These potential drivers may well also be of relevance to the patient, and so justification for a recommendation should be provided.

In addition to a signed consent form, the health professional should also enter a note into the patient's medical records documenting the discussion and resources provided^18^, ^21^ in order to give a meaningful idea of the quality of the consent process.

## Discussion

This review has highlighted the key factors in achieving SDM during the consent process. As suggested by the growing evidence surrounding SDM, the application of this consent framework may improve not only patient satisfaction and decisional conflict but also patient‐reported outcomes and reduce healthcare utilization^7^, ^36^.

Concordance with all factors will take considerable time. Insufficient time is cited as a common clinician‐reported barrier to SDM^9^. Therefore, good‐quality consent must be integrated into clinical practice in a way that will maintain clinical workflow. One potential solution may involve sharing the process between clinicians and other capable team members. Tools such as DAs are likely to play an important role in the future in improving patient understanding and reducing the burden on clinicians. However, it must be recognized that such tools are to promote patient–clinician communication, rather than replacing it.

Another important aspect of engaging clinicians is to ensure that adequate training and guidance are provided. This review provides the structure and description of what should be expected from the consent process; however, practical training and assessments are essential. In this regard, role‐based practical training has been suggested to be effective^4^. Tools to measure the SDM‐centric performance have also been developed to facilitate this^37^, ^38^, but the best strategy remains uncertain.

Patient‐related factors must also be considered when discussing challenges in the practice of SDM. Patients who are lacking knowledge often feel pressure to be passive and compliant^39^. Campaigns such as ‘Ask Share Know’ and ‘Ask Three Questions’ have also been rolled out to prepare patients for such encounters^40^, ^41^.

This review also coincides with plans to update GMC guidance regarding consent. In response, topics in need of further guidance, research and innovation in order to support health professionals and patients in the consent process are highlighted in *Table* 1.

**Table 1:** Suggestions for future development

Suggestion	Description
‘Enhanced’ decision and conversation aids	These should integrate: Novel methods of exploring patient values Core information sets Different sources of outcome data Effective risk communication strategies Novel methods to tailor the content and mode of information delivery
Clinical workflow	Integrating consent conversations into clinical practice in a way that will enhance or maintain workflow
Handling of exceptional rare risks	Determining whether exceptionally rare but serious risks are material to treatment decisions
Involving patients	Developing strategies for improved involvement of patients in SDM
Measuring SDM	There is currently no consensus on how to measure SDM

SDM, shared decision‐making.
